# Residual Renal Function in Children Treated with Chronic Peritoneal Dialysis

**DOI:** 10.1155/2013/154537

**Published:** 2013-11-24

**Authors:** Maria Roszkowska-Blaim, Piotr Skrzypczyk

**Affiliations:** Department of Pediatrics and Nephrology, Medical University of Warsaw, 24 Marszalkowska Street, 00-576 Warsaw, Poland

## Abstract

Residual renal function (RRF) in patients with end-stage renal disease (ESRD) receiving renal replacement therapy is defined as the ability of native kidneys to eliminate water and uremic toxins. Preserved RRF improves survival and quality of life in adult ESRD patients treated with peritoneal dialysis. In children, RRF was shown not only to help preserve adequacy of renal replacement therapy but also to accelerate growth rate, improve nutrition and blood pressure control, reduce the risk of adverse myocardial changes, facilitate treatment of anemia and calcium-phosphorus balance abnormalities, and result in reduced serum and dialysate fluid levels of advanced glycation end-products. Factors contributing to RRF loss in children treated with peritoneal dialysis include the underlying renal disease such as hemolytic-uremic syndrome and hereditary nephropathy, small urine volume, severe proteinuria at the initiation of renal replacement therapy, and hypertension. Several approaches can be suggested to decrease the rate of RRF loss in pediatric patients treated with chronic peritoneal dialysis: potentially nephrotoxic drugs (e.g., aminoglycosides), episodes of hypotension, and uncontrolled hypertension should be avoided, urinary tract infections should be treated promptly, and loop diuretics may be used to increase salt and water excretion.

## 1. Definition and Measurements of Residual Renal Function

Residual renal function (RRF) in patients with end-stage renal disease (ESRD) receiving renal replacement therapy is defined as the ability of native kidneys to eliminate water and uremic toxins. In clinical practice, it is considered synonymous with such parameters as daily diuresis and/or glomerular filtration rate (GFR) [1, 2]. The optimal method to measure RRF has not been established. Most commonly, it is evaluated based on daily diuresis, scaled for body mass or body surface area (BSA) in children [1, 3, 4].

Formulas based on serum creatinine level are used to estimate GFR before initiation of renal replacement therapy. The Schwartz formula [5] or more rarely the Counahan-Barratt equation [6] are used in children and the Modification of Diet in Renal Diseases (MDRD) equation [7] or the Cockcroft-Gault formula [8] in adult patients. This simple approach to evaluate renal function is no longer useful when renal replacement therapy is initiated, as creatinine is also eliminated by dialysis.

According to the National Kidney Foundation Kidney Disease Outcomes Quality Initiative (NKF KDOQI) guidelines, GFR in ESRD patients treated with renal replacement therapy, including pediatric patients, is estimated based on average 24-hour urine creatinine and urea clearance, scaled for patient BSA and expressed in mL/min/1.73 m^2^ or L/week/1.73 m^2^ [1]. Due to problems related to 24-hour urine collection, a search for alternative methods to evaluate RRF continues in clinical studies, including such measurements as serum cystatin C level [9] and renal clearances of iohexol [10] and ^61^Cr-ethylenediaminetetraacetic [11]. Kim et al. showed a significant negative correlation between serum cystatin C level and GFR in children treated with chronic peritoneal dialysis (PD) [9].

### 1.1. Adequacy of Renal Replacement Therapy

The concept of dialysis adequacy was introduced to evaluate the effect of renal replacement therapy on clinical outcomes in patients with ESRD. Adequate dialysis is defined as such amount of dialysis therapy that is sufficient to protect from increased mortality and morbidity [1]. Dialysis adequacy is judged by clinical parameters (patient well-being and lack of uremic symptoms, good nutrition, appropriate blood pressure control, stable body weight, and normal fluid balance and in children also appropriate growth rate and psychosocial development) and laboratory data (appropriate urea, creatinine, electrolyte, albumin, and hemoglobin levels and lack of metabolic acidosis).

This evaluation is routinely combined with measurements of clearance of low-molecular uremic toxins, creatinine and urea. In ESRD patients undergoing renal replacement therapy, these toxins are eliminated by dialysis and with preserved RRF also by native kidneys. Weekly elimination of urea (expressed as total weekly clearance of urea, twKt/V) and creatinine (expressed as total weekly clearance of creatinine, twCCr L/week/1.73 m^2^) is calculated in children according to [12, 13].

These parameters of dialysis adequacy were included in the 1997 NKF KDOQI guidelines. The recommended twKt/V and twCCr values were 2.0 and 60 L/week/1.73 m^2^, respectively, in patients treated with continuous ambulatory peritoneal dialysis (CAPD), 2.1 and 63 L/week/1.73 m^2^ in patients treated with continuous cycling peritoneal dialysis (CCPD), and 2.2 and 66 L/week/1.73 m^2^ in patients treated with nocturnal intermittent peritoneal dialysis (NIPD) [14]. In 2000 Update of NKF KDOQI guidelines twCCr target was lowered for low and low-average transporters in peritoneal equilibration test (PET) treated with CAPD from 60 to 50 L/week/1.73 m^2^. Other targets remained unchanged [15].

The most recent 2006 NKF KDOQI guidelines included also the pediatric population. The recommended twKt/V value in children is, similarly to adult patients, ≥1.8. Based on data from pediatric and adult patients, serum albumin level was found to predict patient survival, and a twKt/V of 1.8 or greater in adult PD patients was associated with improved serum albumin levels [16, 17]. Moreover, the Adequacy of Peritoneal Dialysis in Mexico (ADEMEX) study did not show a benefit associated with twKt/V greater than 1.7 in adult CAPD patients, whereas other studies provided evidence for a recommended minimal twKt/V of greater than 1.7 and an optimal twKt/V of 1.8 based on survival data in anuric adult patients treated with CAPD [18, 19]. No similar large-scale studies have been performed in children, and thus data correlating solute clearance to outcomes cannot be considered definitive. As a result, a twKt/V of 1.8 that is recommended in adults was extrapolated in these guidelines to the pediatric population treated with chronic PD [1].

In the 2006 NKF KDOQI guidelines, twCCr measurements were not recommended to evaluate adequacy of chronic PD [1]. Determination of twKt/V alone currently is recommended for followup based upon the simplicity of the calculation and because studies of adult PD patients have not provided evidence of a benefit in terms of patient outcomes when expressing clearance in any manner other than twKt/V [20, 21].

## 2. Importance of Residual Renal Function in Adult Patients Treated with Peritoneal Dialysis

The initial studied benefit of preserving RRF in adult PD patients was elimination of urea and creatinine which increased total clearance and improved adequacy of renal replacement therapy [22]. RRF loss may result in suboptimal dialysis adequacy, necessitating changes in the PD protocol such as increasing the amount of dialysate fluid used, including high osmolarity fluids, and in some cases combining PD with hemodialysis or switching from PD to an alternative modality of renal replacement therapy [23, 24]. Preserved RRF is particularly important in patients with low peritoneal permeability in peritoneal equilibration test (PET), characterized by low values of peritoneal urea and creatinine clearance [22, 25].

The multicenter Canada-USA Peritoneal Dialysis Study (CANUSA), published in 1997, showed that increasing total urea and creatinine clearance was associated with improved outcomes in adult PD patients [26]. However, Bargman et al. reanalyzed these results and showed a prognostic value of only preserved RRF but not peritoneal clearances. It was estimated that each increase in endogenous creatinine clearance by 5 L/week/1.73 m^2^, corresponding to an approximately 0.5 mL/min/1.73 m^2^ change in GFR, was associated with a reduction of mortality risk by 12% and each increase in urine volume by 250 mL/24 hr was associated with a 36% reduction of mortality risk [27]. These associations were confirmed in multicenter ADEMEX [18] and the Netherlands Cooperative Study on the Adequacy of Dialysis-2 (NECOSAD-2) [28] studies which also showed that not total urea and creatinine clearance but only preservation of RRF and renal clearances had a significant effect on outcomes in adult patients receiving renal replacement therapy.

Compared to anuric patients, those with preserved RRF were shown to be characterized by significantly higher clearances of medium- and high-molecular-weight toxins, for example, organic acids, cystatin C, and *β*2-microglobulin [29, 30], better parameters of calcium-phosphorus balance [31–33], higher hemoglobin levels [32], lower requirement for erythropoiesis stimulating agents (ESA) [33], lower levels of proinflammatory cytokines and inflammation markers such as C-reactive protein [34], better nutrition [32, 35], and improved fluid balance [36]. Anuric PD patients, particularly those with high peritoneal permeability in PET, are at risk of sodium overload and development of hypertension and left ventricular hypertrophy [37] which ultimately increases the risk of cardiovascular events including myocardial infarction and stroke.

### 2.1. Factors Affecting Preservation of Residual Renal Function in Adults

In the recent years, many factors affecting the rate of diuresis loss in adult ESRD patients were identified, for example, the rate of RRF loss in PD patients is 24–80% lower compared to patients on hemodialysis [38–41]. It was suggested, however, that the rate of RRF loss in patients undergoing hemodialysis with the use of ultrapure water, bicarbonate buffer, and high flux polysulfone membranes may be comparable to that in PD patients [42]. Some authors found a significantly faster (*P* < 0.05) rate of RRF loss in automated peritoneal dialysis (APD) patients compared to CAPD patients [43–45], but these findings were not confirmed in other studies [38, 46, 47]. A systematic review of three randomized clinical studies including 139 patients [48–50] also did not show a significant difference in RRF, as measured by GFR, between CAPD and APD groups [51].

In adult patients treated with chronic PD, available literature data do not indicate a clear relation between RRF and the degree of peritoneal permeability. A more rapid RRF loss was observed in patients with both low [52] and high [46] peritoneal permeability, along with a lack of the effect of peritoneal transport characteristics on RRF [43, 53–55]. A high volume of dialysate fluid [54] and increased glucose load [43] were also reported as risk factors for more rapid RRF loss. A slower rate of RRF loss was observed in those adult patients in whom biocompatible dialysate fluids [56] and icodextrin-containing fluid [57] were used.

The effect of blood pressure on RRF in ESRD patients treated with PD is also controversial, with reports of both no effect [46, 47] and an adverse effect of either high [43] or low [53] blood pressure on the native kidney function.

Numerous studies in adult PD patients evaluated the effect of antihypertensive medications (angiotensin-converting enzyme inhibitors, angiotensin receptor blockers, calcium channel antagonists, and loop diuretics) on the rate of RRF loss, but their results are inconsistent [38, 43, 46, 53, 58, 59]. Shemin et al. [60] and Singhal et al. [54] showed a negative effect of aminoglycoside antibiotics used in the treatment of PD-associated peritonitis, but these findings were not confirmed in other studies [61, 62]. An adverse effect of contrast agents was also not clearly shown, provided that dialysis patients are adequately hydrated [63].

The rate of RRF loss may also be related to demographic factors such as age, gender, and ethnicity [38, 43, 46, 47, 54]. Available literature data suggest that the etiology of ESRD has no major effect on preservation of RRF [38], but a more rapid loss of diuresis was observed in patients with severe proteinuria [43, 54, 64]. Other risk factors for RRF loss in adults included diabetes [38, 46, 53, 54] and cardiovascular disease [38, 47, 53].

## 3. Peritoneal Dialysis in Children

Peritoneal dialysis is the treatment of choice as the modality of renal replacement therapy in children with ESRD. Advantages of PD over hemodialysis in pediatric patients are related to a twofold higher peritoneal membrane surface per kilogram of body mass compared to adults, difficulties related to creation and maintenance of adequate vascular access for hemodialysis in the youngest patients, elimination of pain related to punctures of the arteriovenous fistula, and no need for anticoagulant use. A greater degree of patient freedom with this approach allows home dialysis therapy, regular schooling or kindergarten attendance, and engaging in normal everyday life activities [13]. Peritoneal dialysis is the initial approach to renal replacement therapy in 53.1% of children below 15 years of age in Europe [65], 45% of children below 18 years of age in the North America [66], and 39% of children below 18 years of age in Australia and New Zealand [67].

### 3.1. Alterations in Peritoneal Membrane Related to Chronic Peritoneal Dialysis

With a prospect of many years of PD treatment before kidney transplantation, adequate physiology of the peritoneal membrane needs to be maintained for as long as possible.

However, it is now known that multiple adverse morphological changes in the peritoneal membrane including the loss of mesothelium, submesothelial fibrosis, angiogenesis, vasculopathy, and basement membrane duplication occur during PD [68]. Experimental studies showed that the most important factors damaging the peritoneal membrane include episodes of peritonitis and some properties of dialysate fluids such as low pH, high lactate content, high osmolarity, and glucose, glycation degradation products (GDP), and advanced glycation end-products (AGE) including pentosidine, 3-deoxyglucosone, N*ε*-(carboxymethyl)-lysine, or N*ε*-(carboxyethyl)-lysine [69]. These factors increase synthesis of transforming growth factor-*β* (TGF-*β*), activate protein kinase C, stimulate synthesis of reactive oxygen species, and induce activation of local renin-angiotensin-aldosterone (RAA) systems and the leptin pathway in the peritoneal membrane [70]. GDP and AGE are also major stimuli for neoangiogenesis by inducing expression of the most potent proangiogenic factor, vascular endothelial growth factor (VEGF) [71]. Another adverse process in the peritoneal membrane is so called epithelial-to-mesenchymal transformation or the loss of epithelial cell phenotype by mesothelial cells which begin to show myofibroblast properties, with concomitant migration of these cells inside the peritoneal membrane and production of TGF-*β*, VEGF, and extracellular matrix [72].

All these processes impair function of the peritoneal membrane. Fibrosis leads to decreased osmotic conductance, and increased vascularity and vessel permeability results in an increase in the effective peritoneal surface, leading to more rapid glucose absorption from the dialysate fluid [68]. These changes result in the loss of peritoneal ultrafiltration properties which is currently the major problem during long-term PD, occurring in about one third of patients after 4 years of PD [73], being the second most common cause, after infective complications, of the need to give up this approach to renal replacement therapy in children [74, 75].

The most severe form of these negative peritoneal changes during long-term PD is encapsulating peritoneal sclerosis (EPS), found in about 1.5–2.0% of children undergoing chronic PD [76, 77]. The most important risk factors for EPS include long PD duration (≥2 years), frequent severe episodes of peritonitis, use of dialysate fluids with high levels of glucose, GDP, and acetate as the source of bases, and high peritoneal permeability in the initial PET [77, 78].

This inevitable deterioration of peritoneal ultrafiltration properties prompted attempts to develop new approaches to dialysis therapy that would limit these adverse changes in the peritoneal membrane. One important way to limit the use of large amounts of bioincompatible, glucose-rich dialysate fluids and thus extend peritoneal membrane viability and at the same time provide adequate renal replacement therapy is to maintain RRF for as long as possible.

## 4. Importance of Residual Renal Function in Children Treated with Chronic Peritoneal Dialysis

### 4.1. Reduced Levels of Advanced Glycation End-Products

As noted above, one potential benefit of preserved RRF in children receiving chronic PD treatment is longer preservation of ultrafiltration properties of the peritoneal membrane by reduced use of glucose-rich dialysate fluids. In addition, Bayazit et al. found that preserved RRF facilitates removal of AGE in children treated with PD. The authors noted that serum and dialysate fluid level of one AGE, pentosidine, was significantly higher in anuric children compared to those with preserved diuresis (serum level: 24.1 ± 16.6 versus 11.2 ± 8.8 pmol/mg protein, *P* = 0.02; dialysate fluid level: 31.1 ± 3.7 versus 14.9 ± 11.9 pmol/mg protein, *P* = 0.01) [79].

### 4.2. Dialysis Adequacy

Similarly to adult patients, a positive relation was found between RRF and adequacy of renal replacement therapy in children treated with PD [9, 80–83]. All authors point to particularly large difficulties with maintaining clearances of small molecules in children with rapidly decreasing or lost RRF. Both in CAPD [82] and APD [83] patients, strong linear relations were found between residual GFR and volume of diuresis and twKt/V and twCCr (*r* = 0.50–0.92, *P* < 0.05). van der Voort et al. [81], Kim et al. [9], and Montini et al. [84] found that increasing volume and osmolarity of dialysate fluid allowed maintaining normal twKt/V in anuric children treated with chronic PD (CAPD/APD), but twCCr in these patients was significantly lower (*P* < 0.05) compared to children with preserved diuresis. Similarly, in a Finnish study Höltta et al. evaluated dialysis adequacy in 21 children treated with APD for 9 months and found that, with decreasing residual diuresis, twKt/V was maintained at a constant normal level but twCCr decreased [80]. The observed strong relation between twCCr and RRF was highlighted in the 2006 NKF KDOQI guidelines which no longer recommended measuring twCCr as the parameter that is related mostly to RRF and showing only weak association with dialysis treatment, which led to a recommendation of measuring only twKt/V [1].

### 4.3. Growth

In the multicenter Mid-European Pediatric Peritoneal Dialysis Study Group study, Schaeffer et al. evaluated growth rate in 51 children treated with chronic PD and showed a positive correlation between height standard deviation score (SDS) and residual GFR (*r* = 0.34, *P* = 0.01) but found no relation between change in height SDS and RRF during 18 months of followup [85]. In contrast, Chadha et al. showed a relation between growth rate and RRF in a group of 24 American patients. During 12 months of followup, mean height SDS increased in 12 children with preserved diuresis from −1.78 to −1.64 but decreased in 12 anuric children from −1.37 to −1.90 (*P* = 0.01). Moreover, of the 12 patients with RRF, 7 (58%) demonstrated catch-up growth but only 2 (17%) of the 12 patients without RRF. Of note, these authors did not show a relation between growth rate and total creatinine and urea clearances. In contrast, growth rate was related to residual Kt/V (*r* ^2^ = 0.17, *P* = 0.04) and residual creatinine clearance (*r* ^2^ = 0.17, *P* = 0.04) [86]. Similarly, an analysis of 12-month follow-up data in 214 patients in the International Pediatric Peritoneal Dialysis Network (IPPN) database showed that preserved residual diuresis significantly increased the odds of increasing height SDS in children treated with PD (odds ratio: 3.25; 95% confidence interval: 1.66–6.31; *P* < 0.0006) [87].

### 4.4. Nutrition

Preserved RRF not only improves nutrition status but also allows greater freedom when providing nutrition to children with ESRD treated with PD, as highlighted in the 2008 KDOQI guidelines for nutrition in children with chronic kidney disease [88].

In children treated with CAPD/APD, significant positive correlations were found between protein nitrogen appearance (PNA) and residual GFR [83] and renal weekly Kt/V [89] and between daily protein intake (DPI) and renal weekly Kt/V [90]. Edefonti et al. evaluated nutrition status in 43 children treated with APD using an anthropometry-bioimpedance analysis-nutrition (ABN) approach. In this study, malnourished patients were characterized by lower urine volume (343 ± 412 versus 708 ± 587 mL/24 h) and residual GFR (9.8 ± 16.7 versus 15.7 ± 20.6 L/week/1.73 m^2^) although these differences were not significant [91]. However, other authors did not show an association between nutrition status and RRF parameters [92, 93].

### 4.5. Blood Pressure and Cardiovascular Risk

Cardiovascular diseases are the most common cause of death among adults and children with ESRD [94, 95]. Preservation of RRF improves fluid balance, thus reducing cardiovascular load, blood pressure, and cardiovascular risk. Children treated with PD often have hidden fluid overload which increases with deterioration of RRF, leading to increased volume overload, left ventricular hypertrophy, and diastolic heart failure [92].

In a study mentioned above, Bakkaloglu et at. showed negative correlations between urine volume and blood pressures, including systolic (*r* = −0.46, *P* = 0.06), diastolic (*r* = −0.53, *P* < 0.05), and mean blood pressure (*r* = −0.53, *P* < 0.05) [82]. Similarly, systolic and diastolic blood pressure was inversely related to daily diuresis (*r* = −0.38, *P* < 0.05, and *r* = −0.24, *P* < 0.05, resp.) in a Polish multicenter study including children treated with PD or hemodialysis [96].

The purpose of the Turkish multicenter TUPEPD study was to evaluate cardiovascular risk and identify factors affecting cardiac and vascular remodeling in children with ESRD treated with chronic PD. The authors showed that, compared to patients with preserved diuresis, anuric patients were characterized by a significantly greater left ventricular mass index (73 ± 32 versus 52 ± 17 g/m^2^, *P* = 0.009), relative wall thickness (0.53 ± 0.13 versus 0.45 ± 0.11, *P* = 0.025), and more severe diastolic dysfunction as measured by power Doppler early-diastole tissue Doppler imaging (PWD E/TDI) ratio (9.6 ± 3.9 versus 6.9 ± 2.3, *P* = 0.004). In addition, a negative relation between urine volume and left ventricular mass index was found among children with preserved diuresis (*r* = −0.306, *P* = 0.021). Patients with preserved RRF also had higher mean high-density lipoprotein cholesterol level (53 ± 13 versus 42 ± 14 mg/dL, *P* = 0.004). The authors concluded that preservation of RRF in children treated with PD is a key factor for reducing cardiovascular risk and improving outcomes in this patient group [97].

### 4.6. Anemia

Although no studies are available that would clearly show that preserved RRF increases hemoglobin level and facilitates treatment of anemia in children treated with PD, such relationships are suggested by cross-sectional studies. Bakkaloğlu et al. found that daily diuresis was positively correlated with hemoglobin level and hematocrit (*r* = 0.49, *P* < 0.05, and *r* = 0.50, *P* < 0.05, resp.) [82]. Similarly, patients with preserved diuresis in the TUPEPD study had significantly higher hemoglobin levels compared to anuric children (10.8 ± 1.4 versus 8.9 ± 1.6 g/dL, *P* = 0.0001) [97]. Also an analysis of 1394 children treated with chronic PD who were included in the IPPN database showed a negative association between volume of diuresis and hemoglobin level and response to ESA [98]. In contrast, Acar et al. did not show residual GFR to be related to hemoglobin level, hematocrit, and erythropoietin dose in a small group of 24 children treated with PD [92].

### 4.7. Calcium-Phosphorus Balance

Borzych et al. investigated calcium-phosphorus balance parameters in 890 children treated with chronic PD who were included in the international IPPN database. Compared to patients with preserved diuresis, anuric patients were shown to be characterized by significantly higher calcium (2.40 ± 0.24 versus 2.35 ± 0.23 mmol/L, *P* < 0.01) and phosphorus level (1.79 ± 0.42 versus 1.69 ± 0.37 mmol/L, *P* < 0.0001), with no differences in parathormone level. In multivariate analysis, the strongest risk factors of hyperphosphatemia were daily diuresis (*β* = −0.867, *R* ^2^ = 0.023, and *P* < 0.0001) and sexual maturity (*β* = 0.292, *R* ^2^ = 0.008, and *P* < 0.01) [87]. Interesting findings were also reported in the analysis of calcium-phosphorus balance parameters in 51 patients with ESRD treated with PD. The authors showed that serum 25-hydroxyvitamin D level was not related to calcium, phosphorus, and parathormone level but showed an association with weekly residual Kt/V (*r* = 0.385, *P* = 0.005) and creatinine clearance (*r* = 0.443, *P* = 0.001) [99]. Based on these findings, the authors concluded that RRF in children with ESRD treated with PD is one of the key factors maintaining normal calcium-phosphorus balance.

### 4.8. Peritoneal Dialysis-Associated Peritonitis

Boehm et al. attempted to identify risk factors for peritoneal dialysis-associated peritonitis in children based on the analysis of clinical data in 30 children treated with chronic PD (including 21 treated with APD and 9 with CAPD). In univariate analyses, the risk of peritonitis was negatively related to daily diuresis volume (*r* = −0.48, *P* = 0.013) and residual GFR (*r* = −0.54, *P* = 0.012). Similarly, magnitude of RRF along with exit-site infection was the strongest risk factor for peritonitis in the multivariate analysis [83].

## 5. Risk Factors for Residual Renal Function Loss in Children

Compared to adult patients, relatively little is known about factor that might delay RRF loss in children treated with PD.

Feber et al. sought to answer the question whether, similarly to adults, daily diuresis is preserved for a longer time in patients treated with PD compared to those treated with hemodialysis. The authors retrospectively analyzed data on RRF in 28 children treated with hemodialysis and 31 children treated with CAPD during a followup ranging from 6 to 43 months (median 19 months). Longer preservation of daily diuresis and higher mean urine volumes were found in the CAPD group throughout the followup [3].

Fischbach et al. compared preservation of RRF in 60 children treated with hemodiafiltration and 37 children treated with PD (including 32 with APD). The authors showed a significantly higher risk of anuria in the hemodiafiltration group (65% versus 23%, *P* < 0.05) despite the use of biocompatible dialysis membranes and high hemodynamic stability during the procedures. Except for a younger age of children treated with PD, no significant differences were found between the two groups [4].

In a study investigating the use of a biocompatible dialysis fluid with low GDP content in 21 children treated with APD, Schmitt et al. found that residual GFR was stable regardless of the dialysate fluid used [100].

In our retrospective study, we evaluated RRF in 101 children treated with PD (including 44 treated with CAPD and 57 treated with APD). During 3 years of followup, we showed a gradual reduction of daily diuresis and residual GFR, with a significantly higher rate of RRF loss during the first year (*P* < 0.05) among 57 children treated with APD compared to 44 children treated with CAPD and no significant differences in the subsequent years of followup. Also the Kaplan-Meier analysis did not show an increased risk of anuria in the APD group. In our study, the identified risk factors for anuria included hemolytic-uremic syndrome and hereditary nephropathy, low urine volume and residual GFR at the initiation of renal replacement therapy, hypertension, anemia, hypoalbuminemia, hyperlipidemia, and severe proteinuria. In the APD group, the rate of RRF loss was significantly higher among children with low peritoneal permeability (*P* < 0.05). Occurrence of peritonitis, aminoglycoside use, and antihypertensive drug use had no effect on the rate of RRF loss in our study population [101].

## 6. Conclusions

Studies of recent years indicate that preserving RRF has become one of the most important tasks for the nephrologist caring for patients treated with PD. Growing evidence shows benefits from RRF preservation in children, particularly in regard to accelerated growth rate, improved nutrition, and a reduced risk of cardiovascular events.

Based on few studies performed in children, several approaches can be suggested to decrease the rate of RRF loss in pediatric patients treated with chronic peritoneal dialysis [1, 101].

- Potentially nephrotoxic drugs (e.g., aminoglycosides) should be avoided.
- Urinary tract infections should be treated promptly.
- Episodes of hypotension, as well as uncontrolled hypertension, also have a negative effect on RRF and should be avoided.
- Loop diuretics may be used to increase salt and water excretion.

With multiple controversies regarding the use of the RAA system inhibitors in the context of nephroprotection in adult patients treated with PD [59] and the lack of similar studies in children, no clear indications can be established for these drugs in the pediatric population. The choice of antihypertensive agents should be primarily directed at obtaining effective blood pressure control. Close monitoring of serum potassium level is necessary when angiotensin-converting enzyme inhibitors or angiotensin receptor blockers are used in children with ESRD [1].
